# Spectrin-Based Regulation of Cardiac Fibroblast Cell-Cell Communication

**DOI:** 10.3390/cells12050748

**Published:** 2023-02-26

**Authors:** Drew M. Nassal, Rebecca Shaheen, Nehal J. Patel, Jane Yu, Nick Leahy, Dimitra Bibidakis, Narasimham L. Parinandi, Thomas J. Hund

**Affiliations:** 1 The Frick Center for Heart Failure and Arrhythmia, Dorothy M. Davis Heart and Lung Research Institute, The Ohio State University Medical Center, Columbus, OH 43210, USA; 2 Department of Biomedical Engineering, The Ohio State University, Columbus, OH 43210, USA; 3 Department of Internal Medicine, Division of Pulmonary, Critical Care & Sleep Medicine, The Ohio State University, Columbus, OH 43210, USA; 4 Department of Internal Medicine, Division of Cardiovascular Medicine, The Ohio State University, Columbus, OH 43210, USA

**Keywords:** cell-cell communication, cardiac fibroblast, spectrin, STAT3, exosomes

## Abstract

Cardiac fibroblasts (CFs) maintain the fibrous extracellular matrix (ECM) that supports proper cardiac function. Cardiac injury induces a transition in the activity of CFs to promote cardiac fibrosis. CFs play a critical role in sensing local injury signals and coordinating the organ level response through paracrine communication to distal cells. However, the mechanisms by which CFs engage cell-cell communication networks in response to stress remain unknown. We tested a role for the action-associated cytoskeletal protein β_IV_-spectrin in regulating CF paracrine signaling. Conditioned culture media (CCM) was collected from WT and β_IV_-spectrin deficient (*qv^4J^*) CFs. WT CFs treated with *qv^4J^* CCM showed increased proliferation and collagen gel compaction compared to control. Consistent with the functional measurements, *qv^4J^* CCM contained higher levels of pro-inflammatory and pro-fibrotic cytokines and increased concentration of small extracellular vesicles (30–150 nm diameter, exosomes). Treatment of WT CFs with exosomes isolated from *qv^4J^* CCM induced a similar phenotypic change as that observed with complete CCM. Treatment of *qv^4J^* CFs with an inhibitor of the β_IV_-spectrin-associated transcription factor, STAT3, decreased the levels of both cytokines and exosomes in conditioned media. This study expands the role of the β_IV_-spectrin/STAT3 complex in stress-induced regulation of CF paracrine signaling.

## 1. Introduction

Cardiac mechanical function depends on the coordinated activity of cardiac myocytes organized in interconnected muscle fibers and supported by a fibrous extracellular matrix (ECM) maintained by resident cardiac fibroblasts (CFs) [1,2,3]. Under physiological conditions, CFs typically reside in a quiescent (non-activated) state; however, cardiac stress or injury induces a dramatic transition in CF phenotype, characterized by increased proliferation, contractility and excessive ECM production leading to cardiac fibrosis [4,5,6,7]. While cardiac fibrosis is critical for repairing damaged myocardial tissue, dysregulation of the process in disease contributes to cardiac mechanical and electrical dysfunction [3,8,9].

CFs have the remarkable ability to sense local injury signals and communicate distress in a paracrine manner to distal cells, including other CFs, myocytes, immune cells, and endothelial cells [10,11]. Mounting data indicate that CFs engage in a cell-cell communication network following cardiac injury that depends, at least in part, on small (30–150 nm) extracellular vesicles (exosomes) capable of delivering proteins, lipids, mRNA and other bioactive cargo within the heart or even to other organs [12,13,14,15,16,17]. However, the mechanisms responsible for tuning CF exosome-dependent communication networks in response to chronic stress remain unclear. Mechanistic insight into the biogenesis of exosomes and their evolution with disease presents great promise for not only improving existing treatments but expanding therapeutic approaches.

Spectrin family members are actin-associated cytoskeletal proteins that support cellular architecture and membrane stability in metazoan cells [18,19,20]. Beyond mechanical support for the membrane, spectrins facilitate intracellular signaling through the formation of macromolecular complexes involving ion channels, regulatory, adapter molecules, and transcription factors. The β_IV_-spectrin isoform, in particular, has been shown to act as a dynamic scaffold that organizes local signaling domains for regulation of signal transduction events in a variety of cell types, including CFs. Recently, we discovered a novel role for β_IV_-spectrin in regulating the subcellular localization and activity of the pleiotropic transcription factor signal transducer and activator of transcription 3 (STAT3) [21]. Further, we found that disruption of β_IV_-spectrin (in response to chronic stress) promotes STAT3 subcellular redistribution and aberrant activity to alter CF gene expression, proliferation, and contractility [22,23]. At the organ level, β_IV_-spectrin deficiency results in enhance maladaptive remodeling, fibrosis, and cardiac dysfunction, consistent with the CF phenotype [21,22].

Here, we demonstrate that beyond controlling the local activity of individual CFs, β_IV_-spectrin supports a long-range communication network between CFs and other cardiac cells. Using a genetic mouse model of β_IV_-spectrin deficiency (*qv^4J^* mouse), we demonstrate that loss of β_IV_-spectrin triggers the release of paracrine stress signals from CFs in a STAT3-dependent manner with the capacity to alter the behavior of recipient quiescent CFs. Interestingly, we show that β_IV_-spectrin-deficient CFs secrete higher concentrations of both inflammatory paracrine protein factors as well as bioactive exosomes, when compared to quiescent WT CFs. Our work identifies a novel role of the spectrin-based pathway in facilitating long-range communication responsible for impacting healthy cardiac function.

## 2. Materials and Methods

### 2.1. Experimental Animals

Adult (2–4 mos, 18–22 g) male and female C57BL/6J wildtype (WT, control) and β_IV_-spectrin truncated (*qv^4J^*) littermate mice were used (see Table 1 for complete list of abbreviations). *qv^4J^* animals genetically express a *Spnb4* allele with a spontaneous insertion point mutation at C4234T (Q1358 > Stop) resulting in a premature stop codon in β_IV_-spectrin repeat 10 leading to the lack of repeats 11 through the C-terminus including the putative STAT3 binding region [22,24,25]. *qv^4J^* animals were acquired from Jackson Laboratory. All procedures were conducted in accordance with the Guide for the Care and Use of Laboratory Animals published by the National Institutes of Health following protocols approved by the IACUC at The Ohio State University. Animals were euthanized using isoflurane and cervical dislocation followed by collection of tissue or cell isolation.

### 2.2. Isolation of Primary Mouse Cardiac Fibroblasts

Primary mouse CFs were isolated from left and right ventricles under sterile conditions, as described [22,26]. Briefly, ventricular tissue was minced in 2 mg/mL collagenase (Worthington Biochemical, Lakewood, NJ, USA) dissolved in 1× Ham’s F-10 buffer (Corning) at 37 °C. After digestion, the cell extract was filtered and centrifuged. After discarding the supernatant, cells were resuspended in normal feeding media containing 1× DMEM supplemented with 10% FBS, 1% l-glutamine, and 1% Pen/Strep. Cells were plated onto tissue culture treated plates for 4–5 h to allow for adhering. Culture media containing nonadherent cells (e.g., myocytes, endothelial cells) was then removed from culture and discarded. Fresh feeding media was replenished for adhered to CFs. Cells were grown in culture to the desired confluency. All experiments were performed at passage one (P1) conditions [22].

### 2.3. Conditioned Culture Media Collection

After CFs reached ~50–60% confluency, cells were washed with PBS and cultured with exosome depleted FBS (ThermoFisher Scientific, Waltham, MA, USA, Catalog #: A2720803) in DMEM medium with 1% l-glutamine and 1% Pen/Strep for 24 h. CF conditioned culture media (CCM) was collected and centrifuged to remove dead cells/debris. For experiments testing the differences in molecular weight fractions, conditioned media underwent a differential ultracentrifugation process to separate large (includes exosomes) and small (includes proteins and peptides) MW fractions, as described with slight modifications [27,28]. Briefly, CCM was filtered through a 100,000 MW Amicon ultra centrifugal filter unit (Millipore Sigma, Burlington, MA, USA) and centrifuged at 10,000× *g* for 30 min at 4 °C. The flow through was collected as the small MW fraction, while the captured material was resuspended in equal volumes of fresh CCM as the large MW fraction.

### 2.4. CF Proliferation Assay

Separate isolations of WT CFs were seeded into 12-well culture-treated plates, as described [23]. Briefly, cells were adhered for 24 h with serum starvation. The next day medium was replaced with either the complete CCM, small MW media, or large MW media. Cells were trypsinized at 24, 48, and 72 h postplating. Cell pellets were resuspended in a fixed volume and manually counted using a hemacytometer to calculate total cell numbers. Manual counting was performed blindly by the same person throughout the study to maintain accuracy and reproducibility.

### 2.5. Collagen Gel Formation and Macroscopic Gel Contraction Measurements

Type I rat- collagen gels (2 mg/mL) were prepared by mixing 10× PBS, sterile H_2_O, acidic rat tail collagen, and 1 M NaOH. Cells were added (200,000 cells/mL) and mixed before gelation. Cell-collagen mixtures were cast into 24 well culture plates and incubated at 37 °C in 5% CO_2_ for 1 h. After casting, gels were covered with 1 mL of culture feeding media and released from bottom of wells. Photographs of gels following 24 h of incubation were analyzed using NIH ImageJ software (v. 1.53), as described [22]. Experiments were conducted in technical triplicates.

### 2.6. Cytokine/Chemokine Analysis

CCM supernatant from WT and *qv^4J^* CFs was used to examine levels of cytokines/chemokines using Proteome Profiler Mouse XL array kit (R&D Systems, Minneapolis, MN, USA, Catalog #: ARY028) according to manufacturer’s protocol. Briefly, CFs were cultured in normal feeding media until 70–80% confluency was reached. Cells were then serum starved for 24 h. CCM was collected and centrifuged at 2000 rpm for 5 min to remove dead cells/debris. Supernatant was stored at −80 °C until samples were processed and analyzed. Manual cell counts were performed on a subset of experiments at the time of CCM collection. Cytokine/chemokine levels were normalized to a control CC motif chemokine ligand 3 (CCL3).

### 2.7. Characterization and Isolation of Extracellular Vesicles

Isolated CFs from WT and *qv^4J^* mice were grown in culture to ~50–60% confluency (4–5 d after plating). For experiments testing the effects of STAT3 inhibition, a subset of *qv^4J^* CFs were treated for 48 h with S3I-201 (100 µM) [21,22]. Cells were subsequently washed with PBS and cultured with exosome depleted FBS in DMEM medium for 24 h. CCM was collected and centrifuged at 2000× *g* for 30 min to remove any cells or debris. Manual cell counts were performed on a subset of experiments at the time of CCM collection. The supernatant was treated with Total Exosome Isolation reagent, according to the manufacturer’s protocol (0.5× volume of the media, Fisher: 4478359) and incubated overnight at 4 °C. After incubation, the media was centrifuged at 10,000× *g* for 1 h at 4 °C. The supernatant was discarded and the pellet containing the EVs was resuspended in PBS and stored at −80 °C until analysis. EV suspensions were then analyzed for size and count using an NS300 nanoparticle tracking analysis system.

### 2.8. Statistics

Statistical analyses were performed with SigmaPlot 14.5. Data distribution for all comparisons was first tested for normality and equal variance using the Shapiro-Wilk test and Brown-Forsythe test, respectively. For single comparisons, an unpaired two-tailed *t*-test (data presented as mean ± SEM) or Mann-Whitney *U* rank-sum test (data presented as the median with 25th and 75th percentiles [box] and 10th and 90th percentiles [whiskers]) was performed to determine *p* values. For multiple comparisons, a two-way ANOVA with Holm-Sidak post hoc test was used. *p* < 0.05 was determined significant.

## 3. Results

### 3.1. β_IV_-Spectrin Deficiency Alters Cell-Cell Communication in Cardiac Fibroblasts

**Figure 1:**
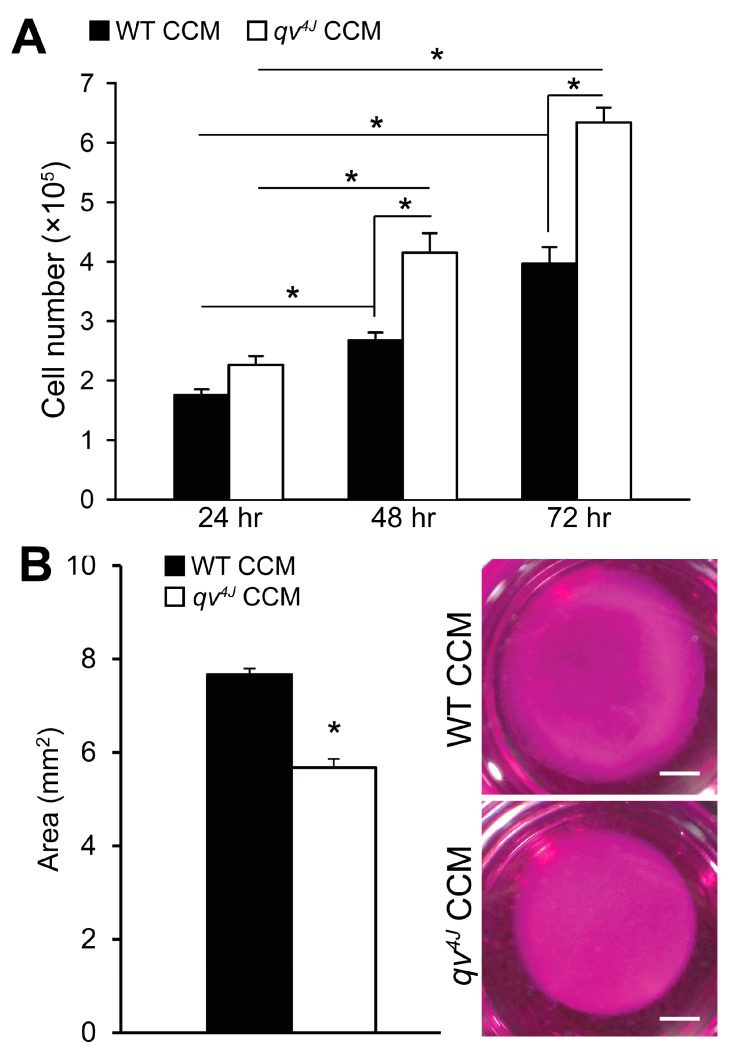
(**A**) Summary data for the number of WT cardiac fibroblasts (CFs) at 24, 48 and 72 h following treatment with either WT or *qv^4J^* culture conditioned media (CCM). All groups had an initial seeding at a density of 1 × 10^5^ cells/well. N = 3 independent preparations; * *p* < 0.05 with two-way ANOVA (with timepoint and genotype as factors) and Holm-Sidak post hoc pairwise comparison. (**B**) Summary data on collagen gel area (**left**) and representative collagen gel images (**right**) following 72 h of treatment of WT CFs with WT or *qv^4J^* CCM. N = 3 independent preparations; * *p* < 0.05 with unpaired two-tailed *t*-test. Scale bars = 2 mm.

To test the hypothesis that β_IV_-spectrin regulates a cell-cell communication network, CFs isolated from adult WT hearts were treated with conditioned culture media (CCM) from CFs isolated from WT (control) or spectrin-deficient hearts (*qv^4J^* mice expressing truncated β_IV_-spectrin). A significant increase in proliferation was observed in WT CFs treated with *qv^4J^* CCM at 48 and 72 h compared to those treated with WT CCM (Figure 1), indicating that spectrin-deficient cells generate paracrine signals capable of altering the phenotype of distal cells.

**Figure 2:**
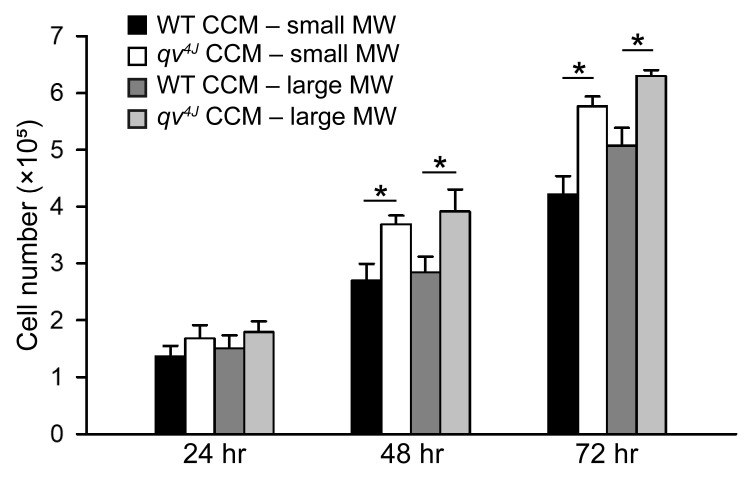
Summary data for the number of WT cardiac fibroblasts at 24, 48 and 72 h following treatment with small MW fraction or large MW fraction of WT or *qv^4J^* culture conditioned media (CCM). All groups had an initial seeding at a density of 1 × 10^5^ cells/well. N = 3 independent preparations; * *p* < 0.05 with two-way ANOVA (with timepoint and CCM group as factors) and Holm-Sidak post hoc pairwise comparison. There was a significant (*p* < 0.05) difference between the 24 h and 48 h timepoints and 48 h and 72 h timepoints for all groups (not indicated in figure for simplicity).

As a first step in identifying β_IV_-spectrin-dependent paracrine signals, WT and *qv^4J^* CF CCM was separated into small and large molecular weight (MW) fractions, with the large MW fraction consisting of exosomes and other extracellular vesicles and the small MW component containing soluble proteins and signaling molecules. Interestingly, both fractions from *qv^4J^* CCM significantly increased proliferation of WT CFs at 48 and 72 h of treatment relative to WT controls, suggesting that β_IV_-spectrin modulates multiple targets relevant for cell-cell communication (Figure 2).

### 3.2. β_IV_-Spectrin Deficiency Alters Secretion of Pro-Inflammatory and Pro-Fibrotic Cytokines/Chemokines from Cardiac Fibroblasts

**Figure 3:**
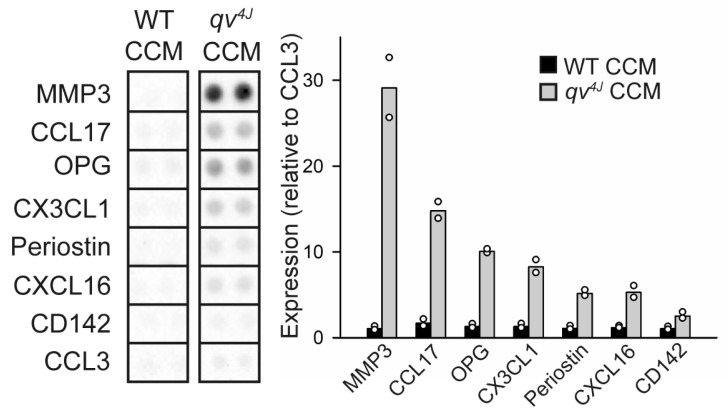
Representative dot blots (noncontiguous images from 2 separate assays for WT and *qv^4J^*) and quantitative estimation on top candidates from proteome profiler mouse cytokine array performed on conditioned culture media (CCM) from WT and *qv^4J^* cardiac fibroblasts following 24 h in serum-free media. Quantitative estimation was performed by normalizing densitometry value for each sample to a control [CC motif chemokine ligand 3 (CCL3)] and then expressing as percent change in *qv^4J^* compared to WT CCM. N = 2 independent preparations for each condition; Bars indicate mean value (data points superimposed). Abbreviations are as follows: CCL17 = CC motif chemokine ligand 17; CD142 = coagulation factor III; CX3CL1 = C-X3-C motif chemokine ligand 1 (or fractalkine); CXCL16 = C-X-C motif chemokine ligand 16; MMP3 = matrix metallopeptidase 3; OPG = osteoprotegerin.

To identify specific paracrine factors secreted by β_IV_-spectrin-deficient CFs to alter the behavior of distal cells, a proteome profiler array assay was used to screen 111 cytokines/chemokines in CCM from WT and *qv^4J^* CFs. Increased expression of a host of pro-fibrotic and pro-inflammatory factors was observed in *qv^4J^* CCM compared to WT, including MMP3, periostin, CCL17, osteoprotegerin, and CX3CL1 (Figure 3), consistent with previous RNA-sequencing analysis showing significant upregulation of these factors at the gene level within *qv^4J^* derived CFs [22]. Although *qv^4J^* CFs show enhanced proliferation, which on its own could increase generation of paracrine factors due to larger cell numbers, the levels of cytokines/chemokines and exosomes were assessed in CCM collected only 24 h after the cells reached target confluency, which is not enough time for differences in proliferation rate to confound the results (compare cell numbers in WT and *qv^4J^* at 24 h timepoint in Figure 1). These data indicate that loss of β_IV_-spectrin triggers the release of a host of cytokines and chemokines with the capacity to modulate phenotype of neighboring cells.

### 3.3. Exosomes Contribute to β_IV_-Spectrin Dependent Cell-Cell Communication

**Figure 4:**
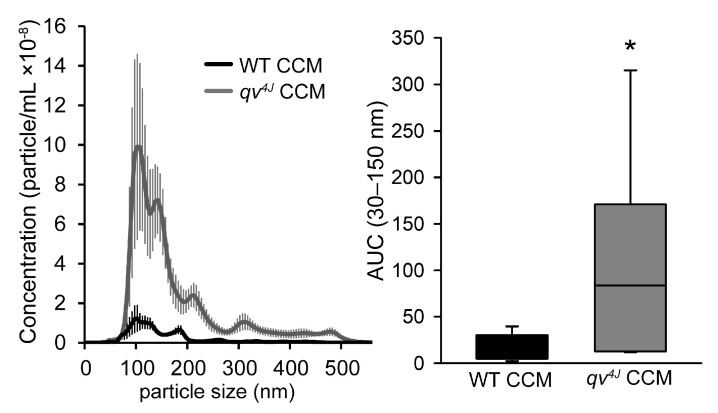
Size distribution of extracellular vesicles isolated from WT or *qv^4J^* conditioned culture medium (CCM) (**left**) and summary data (**right**) on area under the curve (AUC) in the 30–150 nm size range. N = 7 independent preparations for each condition; * *p* < 0.05 vs. WT with Whitney-Mann rank-sum test.

To further explore the role of β_IV_-spectrin in exosome biogenesis/secretion, exosomes from WT and *qv^4J^* CFs were isolated and characterized. The size distribution and number of isolated exosomes were quantified using a NanoSight Particle Tracking system (Nanosight300). This approach confirmed that WT and *qv^4J^* CCM was enriched in extracellular vesicles within the size range that is characteristic for exosomes (Figure 4, 30–150 nm). Consistent with the increased cell-cell communication from spectrin deficient CFs, the concentration of exosomes was significantly greater in *qv^4J^* CCM compared to WT CCM (Figure 4), despite similar CF numbers at time of collection (compare cell numbers in WT and *qv^4J^* at 24 h timepoint in Figure 1).

### 3.4. Role of STAT3 in β_IV_-Spectrin Dependent Cell-Cell Communication

**Figure 5:**
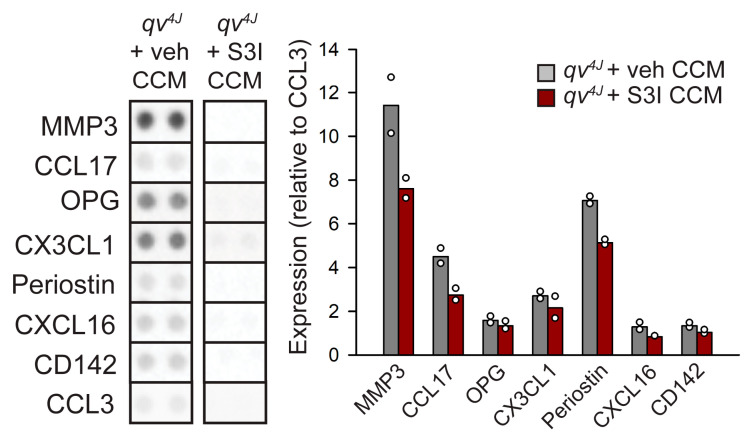
Dot blots (noncontiguous images from 2 separate assays for vehicle and S3I-201) from proteome profiler mouse cytokine array performed on conditioned culture media (CCM) from *qv^4J^* cardiac fibroblasts pretreated for 72 h with STAT3 inhibitor S3I-201 (100 μM) or vehicle control (N = 2 independent preparation for each condition). Abbreviations are as follows: CC motif chemokine ligand 3 = CCL3; CCL17 = CC motif chemokine ligand 17; CD142 = coagulation factor III; CX3CL1 = C-X3-C motif chemokine ligand 1 (or fractalkine); CXCL16 = C-X-C motif chemokine ligand 16; MMP3 = matrix metallopeptidase 3; OPG = osteoprotegerin.

β_IV_-spectrin alters CF gene transcription (including for several targets identified here, Figure 2) in a STAT3-dependent manner [22]. To determine whether altered STAT3 activity contributes to release of proinflammatory and profibrotic cytokines/chemokines from β_IV_-spectrin-deficient CFs, *qv^4J^* CFs were pre-treated with the STAT3 inhibitor S3I-201 (100 µM) or vehicle (3% DMSO in PBS) for 72 h before collection and analysis of CCM. Interestingly, STAT3 inhibition largely normalized the profile of secreted chemokines/cytokines in *qv^4J^* to that observed in WT CCM (Figure 5).

**Figure 6:**
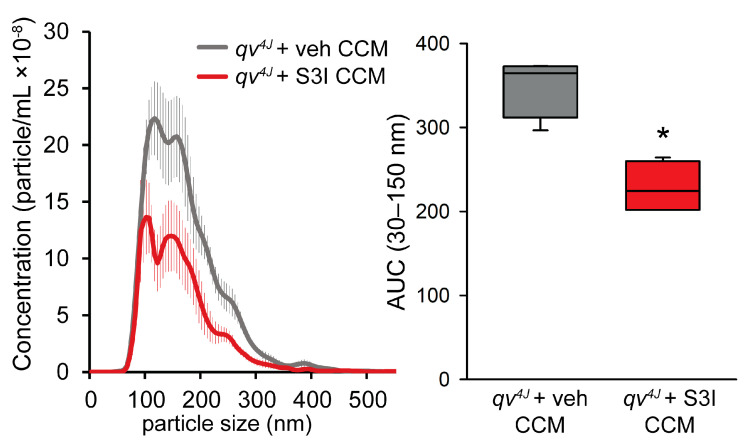
Size distribution of extracellular vesicles isolated from conditioned culture medium (CCM) collected from *qv^4J^* fibroblasts ± STAT3 inhibitor S3I-201 (100 M) (**left**) and summary data (**right**) on area under the curve (AUC) in the 30–150 nm size range. N = 4 independent preparations for each condition; * *p* < 0.05 with Whitney-Mann rank-sum test.

STAT3 inhibition also reduced the concentration of exosomes secreted by *qv^4J^* CFs (Figure 6). These data suggest that β_IV_-spectrin regulates CF paracrine signaling in a STAT3-dependent manner.

## 4. Discussion

Here, we describe a novel role for β_IV_-spectrin in tuning a cell-cell communication network in heart. Specifically, we report that β_IV_-spectrin-deficient (*qv^4J^*) CFs secrete a host of pro-fibrotic and pro-inflammatory paracrine signals into CCM with the capacity to alter the proliferative activity of WT CFs. Furthermore, we demonstrate an increase in release of bioactive exosomes from *qv^4J^* CFs. Finally, we report that STAT3 inhibition normalized the secretory profile of *qv^4J^* CFs. Based on our findings, we propose that the β_IV_-spectrin/STAT3 axis serves as a new avenue for modulating cell-cell communication and cardiac function in the setting of chronic disease.

Degradation of β_IV_-spectrin induces changes in STAT3 signaling and gene expression in both cardiomyocytes and CFs that drive altered cardiac function and fibrosis. At the cellular level, β_IV_-spectrin/STAT3 dysfunction promotes highly eccentric cardiomyocyte growth and increased collagen deposition, proliferation, and contractility in CFs. A similar phenotype is observed in *qv^4J^* mice. Further, the distinctive remodeling profile of hypertrophy and fibrosis was observed together even in cardiomyocyte- or fibroblast-specific β_IV_-spectrin knock out models, despite confirmation of β_IV_-spectrin expression in Cre negative cells [21,22]. Interestingly, global CF activation has been observed following ischemic injury, even in remote areas from the infarct region, although the mechanism for propagating the pro-fibrotic stimuli is unknown [29,30]. Our new findings reveal a potential mechanism for how those pathological changes are communicated throughout the myocardium. Together, these studies implicate a role for the β_IV_-spectrin/STAT3 complex in cell-cell communication. It will be important for future studies to test the role for this complex in cell-cell communication in vivo. In this context, we have the ability to home in on specific cell populations (e.g., CFs, myocytes, immune cells) using our cell-specific β_IV_-spectrin knockout model [21,22].

Over the last decade, there has been growing interest in the characteristics and functional effects of cardiac cell-derived exosomes in the setting of pathologic stress. Many studies have described specific miRNA expression changes in secreted exosomes following pathological stress. For example, increased circulating exosomes enriched with miR-22 have been reported following ischemic injury and proposed to aide in repair and remodeling following myocardial infarction [31]. However, the mechanisms underlying these pathologic changes in exosome secretion are not well defined. Here, we report that β_IV_-spectrin-deficiency increases the secretion of exosomes from CFs. Exosomes have an endosomal origin and are thought to be trafficked using the same pathways as exocytosis and endocytosis. The secretion of extracellular vesicles relies on the highly dynamic membrane-cytoskeletal interface [32,33]. In response to internal and external stimuli, cytoskeletal proteins undergo localized remodeling that results in detachment from the membrane, exposing locations for fusion and vesicle secretion [34,35,36]. Interestingly, it is thought that the actin-spectrin network can regulate not only the location of vesicle secretion, but the dynamics as well [34,37]. For example, in neurons, F-actin regulates the pore opening and speed of vesicle secretion [38,39]. Further, other studies have speculated that spectrin proteins can modulate actin assembly dynamics [20,40]. While it is known that β_IV_-spectrin associates with F-actin at the cell membrane, it is still unclear whether the dissociation of the β_IV_-spectrin-complex from F-actin directly alters the secretion of exosomes. Although we did not directly test this relationship, it will be interesting in the future to determine whether spectrin-dependent regulation of exosome secretion depends on β_IV_-spectrin/F-actin interaction.

STAT3 has a multifaceted role in regulating cell-cell communication, cardiac inflammation, and fibrosis. In CFs, STAT3 and miR-21 form a positive feedback loop to increase proliferation and expression of fibrotic genes, while STAT3 inhibition leads to the downregulation of miR-21 and abrogated myofibroblast activation fibrosis [41]. Interestingly, miR-21 was also found to be abundant in cardiac fibroblast-derived exosomes [14]. This may explain the observed decrease in exosome production following STAT3 inhibition. Our findings add to the growing literature that STAT3 is a critical regulator of cell communication during disease. Importantly, our results demonstrate that the β_IV_-spectrin/STAT3 complex plays a role in exosome biogenesis.

Cardiac wound healing (e.g., following myocardial infarction) relies on paracrine signaling between different cell types to carefully orchestrate the transition from inflammation to repair. Mounting data support the idea that CFs have a role in initiating the inflammasome. Here, we found that loss of β_IV_-spectrin lead to increased expression of pro-reparative and pro-inflammatory stimuli. For example, we observed increased expression of CCL17 and CXC3CL1, chemokine ligands that play a vital role in immune cell infiltration [42,43]. Further, we found increased expression of ECM-degrading matrix metalloproteinases (MMP2 and MMP3) and OPG, which are secreted from CFs to aide in repair of the myocardium [44,45]. The generation of these paracrine signals was abrogated with STAT3 inhibition and reintroduction of the full β_IV_-spectrin construct. These findings support our previous work that loss of β_IV_-spectrin and STAT3 dysregulation is a critical step for CF activation and communication.

While our findings demonstrate regulatory capacity for the β_IV_-spectrin/STAT3 complex in exosome production, our study focuses on the communication between CFs. Going forward, it will be interesting to determine whether a similar pathway supports communication between CFs and other cardiac cells, like cardiomyocytes and immune cells and to assess its role in vivo using cell-specific β_IV_-spectrin knockout models. It will also be interesting to determine how β_IV_-spectrin links specific stress stimuli to changes in exosome production and/or cargo. We propose that stress-induced loss of β_IV_-spectrin not only triggers activation of CFs, but also initiates pathological signal generation that is required for remodeling.

## 5. Conclusions

Overall, this study expands the role of the β_IV_-spectrin/STAT3 complex in mediating cell-cell communication. We found that β_IV_-spectrin deficiency in CFs resulted in increased secretion of cytokines and exosomes that induced phenotypic changes (increased proliferation and contractility) in quiescent CFs. The secretory profile of β_IV_-spectrin deficient CFs could be attenuated by inhibiting STAT3. Our findings implicate potential mechanisms as to how CFs modulate exosome secretion following chronic stress. Future work will dissect the interplay of these interactions in exosome biogenesis and secretion. It will be exciting to validate the contribution of the β_IV_-spectrin/STAT3 complex in cell communication in future in vivo studies.

## Figures and Tables

**Table 1:** Table of abbreviations.

Abbreviation	Definition
CCL17	CC motif chemokine ligand 17
CCL3	CC motif chemokine ligand 3
CCM	Conditioned culture media
CD142	Coagulation factor III
CF	Cardiac fibroblast
CX3CL1	C-X3-C motif chemokine ligand 1
CXCL16	C-X-C motif chemokine ligand 16
ECM	Extracellular matrix
EV	Extracellular vesicle
MMP3	Matrix metallopeptidase 3
MW	Molecular weight
OPG	Osteoprotegerin
STAT3	Signal transducer and activation of transcription 3
*qv^4J^*	Mouse with point mutation in *Spnb4* gene resulting in truncated β_IV_-spectrin
WT	Wildtype

## Data Availability

Not applicable.
